# Performance of D-dimer to lymphocyte ratio in predicting the mortality of COVID-19 patients

**DOI:** 10.3389/fcimb.2022.1053039

**Published:** 2022-12-15

**Authors:** Fei Peng, Qiong Yi, Quan Zhang, Jiayi Deng, Cheng Li, Min Xu, Chenfang Wu, Yanjun Zhong, Shangjie Wu

**Affiliations:** ^1^ Department of Respiratory Medicine, The Second Xiangya Hospital of Central South University, Changsha, China; ^2^ Critical Care Medicine, The Second Xiangya Hospital of Central South University, Changsha, China; ^3^ Department of respiratory medicine, Hunan Provincial People’s Hospital, Changsha, China

**Keywords:** COVID-19, D-dimer, lymphocyte, D-dimer to lymphocyte ratio, mortality, immunothrombosis

## Abstract

**Background:**

Nowadays, there is still no effective treatment developed for COVID-19, and early identification and supportive therapies are essential in reducing the morbidity and mortality of COVID-19. This is the first study to evaluate D-dimer to lymphocyte ratio (DLR) as a prognostic utility in patients with COVID-19.

**Methods:**

We retrospectively analyzed 611 patients and separated them into groups of survivors and non-survivors. The area under the curve (AUC) of various predictors integrated into the prognosis of COVID-19 was compared using the receiver operating characteristic (ROC) curve. In order to ascertain the interaction between DLR and survival in COVID-19 patients, the Kaplan-Meier (KM) curve was chosen.

**Results:**

Age (*OR* = 1.053; 95% *CI*, 1.022-1.086; *P* = 0.001), NLR (*OR* = 1.045; 95% *CI*, 1.001-1.091; *P* = 0.046), CRP (*OR* = 1.010; 95% *CI*, 1.005-1.016; *P* < 0.001), PT (*OR* = 1.184; 95% *CI*, 1.018-1.377; *P* = 0.029), and DLR (*OR* = 1.048; 95% *CI*, 1.018-1.078; *P* = 0.001) were the independent risk factors related with the mortality of COVID-19. DLR had the highest predictive value for COVID-19 mortality with the AUC of 0.924. Patients’ survival was lower when compared to those with lower DLR (Log Rank *P <*0.001).

**Conclusion:**

DLR might indicate a risk factor in the mortality of patients with COVID-19.

## Introduction

After being discovered for the first time in Wuhan, China in December 2019, the coronavirus disease 2019 (COVID-19) moved across the globe at an unprecedented speed. The World Health Organization (WHO) declared it to be a global pandemic on March 11, 2020, which marked the outbreak of COVID-19 in the world (Chen et al., 2021). The effects of COVID-19 on health and socio-economic development are still being felt and no viable remedy has yet been identified. Almost 5% of critically ill patients exhibit a rapid progression to acute respiratory distress syndrome (ARDS), which ultimately results in functional failure of important organs or even death with a mortality rate of 49% among critical cases, despite the fact that the majority of COVID-19 patients are asymptomatic or have mild symptoms (Wu and McGoogan, 2020). Early detection and supportive therapy can significantly lower morbidity and mortality. Therefore, timely establishment of reliable predictors associated with the prognosis of COVID-19 is of paramount importance.

The most sensitive coagulation measure, fibrin fragment D-dimer, which is formed when plasmin cleaves the insoluble fibrin, predicts higher thrombosis risk and frequently serves as a rule-out method for thrombosis with a high sensitivity of 80%−100% and negative predictive value (NPV) of up to 100% (Wells et al., 2001). Nonetheless, D-dimer has limited specificity for vascular embolism, and an increase in D-dimer level appears to occur when there is a production or breakdown of fibrin. According to reports, the D-dimer level is enhanced in several different circumstances, including infectious disorders, pregnancy, cancer, trauma, and surgery (Snijders et al., 2012; Lippi et al., 2014; Fruchter et al., 2015). The most common cause of an increase in D-dimer levels in adult patients receiving emergency care is infection, not venous thrombosis (Lippi et al., 2014). Coagulation and inflammation have a mutually beneficial relationship. The coagulation system can be activated by the released inflammatory mediators and, in turn, some coagulation components can intensify inflammatory responses (Marshall, 2001).

The immune system is maintained by lymphocytes and the subsets that include B cells, natural killer (NK) cells, CD4+ T cells, and CD8+ T cells. Mature naive T cells with antigen experience can transform into central and effector memory cells (van den Broek et al., 2018). The central memory cells can be activated by the contact of another antigen and developed into central or effector memory cells, while the important function of effector T lymphocytes is the clearance of virus-infected cells (Boldt et al., 2014), which is the primary task of T CD8+ cells. Mature CD4+ cells stimulate the development of B lymphocytes to produce specific antibodies and control the proliferation, activation, and differentiation of CD8+ cytotoxic T lymphocytes. Variable virus types have different numbers of total lymphocytes and subsets, indicating a possible connection between viral pathogenesis processes and changes in lymphocyte subsets (Li et al., 2004). Numerous studies have demonstrated that the majority of COVID-19 patients had peripheral blood lymphocyte counts and subsets that were significantly lower than normal, particularly in the severe cases (Wang et al., 2020), which were previously reported and confirmed in severe acute respiratory syndrome (SARS) and Middle East respiratory syndrome (MERS) (Huang et al., 2005; Chu et al., 2016).

However, there is currently no published literature examining the association of D-dimer to lymphocyte ratio (DLR) with the prognosis of COVID-19. Thus, the goal of the current study was to determine whether DLR might be used as a biomarker to predict COVID-19 prognosis.

## Materials and methods

### Study design and participants

The real-time polymerase chain reaction was used to diagnose 611 patients with COVID-19 who had been hospitalized by March 26, 2020, at Tongji Medical College of Huazhong University of Science and Technology and Public Health Treatment Center of Changsha, China. This was a retrospective study involving multiple centers. Patients aged 18 years or older were recruited for the study. With regard to survival status, we split the patients into two groups: survivors and non-survivors. The study was approved by the Ethics Committee of Second Xiangya Hospital of Central South University (No.2020001).

### Data collection

On admission, routine blood test results (white blood cells [WBC], lymphocytes, neutrophils, neutrophils to lymphocytes ratio [NLR], platelet [PLT], C-reactive proteins [CRP], prothrombin time [PT], activated partial thromboplastin time [APTT], D-dimer, and DLR) were noted along with demographic information and symptoms of underlying disease.

### Definition and endpoint

The analysis of the receiver operating characteristic (ROC) curve for the effect of DLR in predicting the mortality of COVID-19 defined high (or low) DLR as the ratio above or equal (or below) the cutoff value. Patient deaths while being treated in a hospital served as the endpoint.

### Statistical analysis

Continuous variables were expressed as the median with interquartile range (IQR), and due to the non-normal distribution of all the data, the Mann-Whitney test was used to examine differences between survivors and non-survivors. For categorical variables, frequency and percentage were given, and Fisher’s exact test (χ^2^ test) was used to assess, as necessary. The risk factors linked to the mortality of COVID-19 patients were screened using a univariate and multivariate logistic regression. To determine the predictive value of biomarkers for the mortality of COVID-19, the ROC curve and the AUC were examined. Survival analysis with high and low levels of DLR was carried out utilizing the Kaplan-Meier (KM) curve and the log rank test. IBM^®^SPSS software, version 26 (Chicago, IL, USA) was used for all statistical analyses.

## Results

A total of 611 COVID-19 patients were enrolled in the research, of whom 537 recovered and 74 died. Demographic characteristics, symptoms, and comorbidities of all patients are shown in **Table 1**. The median ages of the survivors and non-survivors are 54.50 years and 70 years, respectively. Comparing non-survivors with survivors, there were statistically significant variations in the age, gender, and cardiovascular disease comorbidity (*p* < 0.05).

**Table 1:** Baseline characteristics of survivor and non-survivor of COVID-19 patients.

	Total	Survivor (n = 537)	Non-survivor (n = 74)	*P* value
Age,years	57.00 (41.00-68.00)	54.50 (40.25-67.00)	70.00 (62.75-75.25)	**<0.001**
Gender (n,%) Male Female	314 (51.4) 297 (48.6)	267 (49.7) 270 (40.3)	47 (63.5) 27 (36.5)	**0.019**
Symptoms				
Fever (n,%)	480 (78.6)	418 (77.8)	62 (83.8)	0.243
Cough(n,%)	469 (76.8)	410 (76.4)	59 (79.7)	0.519
Myalgia (n,%)	98 (16.0)	82 (15.3)	16 (5.6)	0.521
Fatigue (n,%)	239 (39.1)	202 (37.6)	37 (21.6)	0.163
Headache (n,%)	82 (13.4)	70 (13.0)	12 (16.2)	0.452
Diarrhoea (n,%)	148 (24.2)	129 (24.0)	19 (25.7)	0.756
Abdominal pain (n,%)	31 (5.1)	25 (4.7)	6 (8.1)	0.205
Shortness of breath (n,%)	193 (31.6)	163 (30.4)	30 (40.5)	0.064
Comorbidities				
Hypertension (n,%)	173 (28.3)	145 (27.0)	28 (37.8)	0.053
Cardiovascular disease (n,%)	51 (8.3)	36 (6.7)	15 (20.3)	**<0.001**
Diabetes (n,%)	101 (16.5)	83 (15.5)	18 (24.3)	0.054
Cerebrovascular disease (n,%)	25 (4.1)	19 (3.5)	6 (8.1)	0.063

COVID-19, Coronavirus disease 2019.

*P* values indicate differences between survivor and non-survivor of COVID-19 patients. *P*<0.05 was considered statistically significant. Statistically significant values are showed in bold.

Regarding laboratory findings on admission, the levels of white blood cells (WBC), neutrophils, neutrophils to lymphocytes ratio (NLR), C-reactive proteins (CRP), prothrombin time (PT), activated partial thromboplastin time (APTT), D-dimer, and DLR of the non-survivors were significantly higher compared with the survivors (*p* < 0.05), whilst the levels of lymphocytes, platelet, and albumin of the non-survivors were significantly lower than those of the survivors (*p* < 0.05) (**Table 2**).

**Table 2:** Laboratory findings between the survivor and non-survivor of COVID-19 patients.

	Total	Survivor (n = 537)	Non-survivor (n = 74)	*P* value
WBC, ×10^9^/L	5.42 (4.01-7.44)	5.14 (3.90-6.82)	9.81 (6.24-13.57)	**<0.001**
Lymphocytes, ×10^9^/L	1.03 (0.72-1.53)	1.12 (0.80-1.59)	0.57 (0.40-0.73)	**<0.001**
Neutrophils, ×10^9^/L	3.50 (2.45-5.67)	3.31 (2.39-4.88)	8.76 (5.24-12.63)	**<0.001**
NLR	3.07 (1.94-6.72)	2.70 (1.83-5.01)	13.32 (7.73-29.61)	**<0.001**
PLT,×10^9^/L	181.00 (138.00-246.00)	183.00 (142.25-246.75)	162.50 (103.00-222.50)	**0.002**
CRP, mg/L	19.30 (4.00-57.90)	15.65 (3.80-43.00)	92.60 (55.28-177.08)	**<0.001**
ESR,mm/h	39.00 (19.00-65.00)	39.00 (19.25-65.75)	43.50 (16.75-64.00)	0.947
Albumin,mg/L	36.52 (32.80-40.18)	36.92 (33.50-40.10)	30.50 (27.63-33.70)	**<0.001**
PT,sec	13.3 (11.90-14.30)	12.90 (11.70-14.00)	15.20 (14.00-18.10)	**<0.001**
APTT,sec	35.60 (31.78-40.40)	35.30 (31.60-39.50)	39.65 (34.28-46.23)	**<0.001**
D-dimer,μg/L	0.50 (0.22-1.45)	0.40 (0.21-0.97)	5.04 (1.53-21.00)	**<0.001**
DLR	0.47 (0.17-1.72)	0.36 (0.15-1.04)	12.29 (2.73-32.39)	**<0.001**

COVID-19, Coronavirus disease 2019; WBC, White blood cells; NLR, neutrophils-to-lymphocytes ratio; PLT, platelet; CRP, C-reactive proteins; ESR, Erythrocyte sedimentation rate; PT, prothrombin time; APTT, activated partial thromboplastin time; DLR, D-dimer-to-lymphocytes ratio.

*P* values indicate differences between survivor and non-survivor of COVID-19 patients. *P*<0.05 was considered statistically significant. Statistically significant values are showed in bold.

The risk variables related to the mortality of patients with COVID-19 were age, gender, comorbidity with cardiovascular disease, WBC, lymphocytes, neutrophils, NLR, PLT, CRP, PT, APTT, D-dimer, and DLR (*p* < 0.05) (**Table 3**).

**Table 3:** Univariate analysis of risk factors related to the mortality of COVID-19 patients.

Variables	Odds Ratio (95% CI)	*P* value
Age,years	1.075 (1.053-1.098)	**<0.001**
gender	0.547 (0.329-0.909)	**0.020**
Cardiovascular disease	3.538 (1.829-6.845)	**<0.001**
WBC, ×10^9^/L	1.207(1.145-1.273)	**<0.001**
Lymphocytes, ×10^9^/L	0.028 (0.011-0.070)	**<0.001**
Neutrophils, ×10^9^/L	1.247 (1.178-1.320)	**<0.001**
NLR	1.120 (1.089-1.151)	**<0.001**
PLT,×10^9^/L	0.995 (0.991-0.998)	**0.001**
CRP, mg/L	1.020 (1.016-1.025)	**<0.001**
ESR,mm/h	1.000 (0.992-1.009)	0.986
PT,sec	1.677 (1.479-1.901)	**<0.001**
APTT,sec	1.090 (1.058-1.122)	**<0.001**
D-dimer,μg/L	1.203 (1.157-1.250)	**<0.001**
DLR	1.117(1.089-1.145)	**<0.001**

COVID-19, Coronavirus disease 19; CI, confidence interval; WBC, White blood cells; NLR, neutrophils-to-lymphocytes ratio; PLT, platelet count; CRP, C-reactive proteins; ESR, Erythrocyte sedimentation rate; PT, prothrombin time; APTT, activated partial thromboplastin time; DLR, D-dimer-to-lymphocytes ratio. *P* < 0.05 was considered statistically significant. Statistically significant values are showed in bold.

Age (*OR* = 1.053; 95% *CI*, 1.022−1.086; *p* = 0.001), NLR (*OR* = 1.045; 95% *CI*, 1.001−1.091; *p* = 0.046), CRP (*OR* = 1.010; 95% *CI*, 1.005−1.016; *p* < 0.001), PT (*OR* = 1.184; 95% *CI*, 1.018−1.377; *p* = 0.029), and DLR (*OR* = 1.048; 95% *CI*, 1.018−1.078; *p* = 0.001) were the independent risk factors related with the mortality of COVID-19 (**Table 4**).

**Table 4:** Multivariate analysis of risk factors related to the mortality of COVID-19 patients.

Variables	B	SE	Wald	OR(95% CI)	*P* value
gender	-0.158	0.372	0.180	0.854 (0.412-1.770)	0.671
Age,years	0.052	0.016	11.101	1.053 (1.022-1.086)	**0.001**
Cardiovascular disease	0.082	0.523	0.025	1.086 (0.390-3.027)	0.875
WBC, ×10^9^/L	-0.009	0.048	0.036	0.991 (0.901-1.089)	0.849
NLR	0.044	0.022	3.964	1.045 (1.001-1.091)	**0.046**
PLT,×10^9^/L	0.001	0.002	0.005	1.000 (0.996-1.004)	0.941
CRP, mg/L	0.010	0.003	13.667	1.010 (1.005-1.016)	**<0.001**
PT,sec	0.169	0.077	4.793	1.184 (1.018-1.377)	**0.029**
APTT,sec	0.033	0.021	2.511	1.033 (0.992-1.076)	0.113
DLR	0.047	0.015	10.136	1.048 (1.018-1.078)	**0.001**

COVID-19, Coronavirus disease 19; SE, standard error; OR, odds ratio; CI, confidence interval; WBC, White blood cells; NLR, neutrophils-to-lymphocytes ratio; PLT, platelet count; CRP, C-reactive proteins; PT, prothrombin time; APTT, activated partial thromboplastin time; DLR, D-dimer-to-lymphocytes ratio.

*P* < 0.05 was considered statistically significant. Statistically significant values are showed in bold.

**Figure 1:**
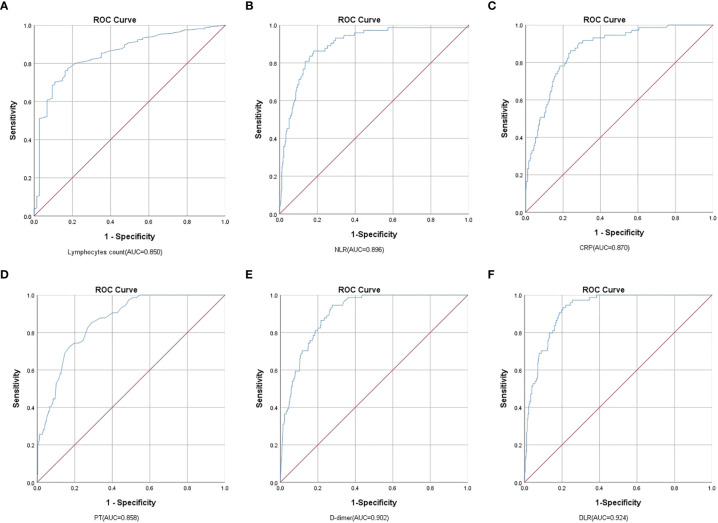
Analysis of the ROC curve of the **(A)** Lymphocytes, **(B)** NLR, **(C)** CRP, **(D)** PT, **(E)** D-dimer, and **(F)** DLR for the mortality of COVID-19. Comparing Lymphocyte, NLR, CRP, PT, D-dimer, and DLR, DLR had the highest AUC in predicting COVID-19 mortality.

ROC analysis indicated that lymphocytes, NLR, CRP, PT, D-dimer, and DLR were able to significantly predict the mortality of patients with COVID-19 (*p* < 0.001) with the AUCs being 0.850 (95% *CI*, 0.820−0.878), 0.896 (95% *CI*, 0.869−0.919), 0.870 (0.840−0.896), 0.858 (95% *CI*, 0.828−0.885), 0.902 (95% *CI*, 0.875−0.924), and 0.924 (95% *CI*, 0.900−0.943), respectively. The cutoff values for lymphocytes, NLR, CRP, PT, D-dimer, and DLR were 0.78 (sensitivity, 83.78; specificity, 75.98), 6.28 (sensitivity, 86.30; specificity, 81.94), 42.30 (sensitivity, 86.30; specificity, 76.00), 13.80 (sensitivity, 85.14; specificity, 71.08), 0.82 (sensitivity, 94.59; specificity, 72.20), and 1.29 (sensitivity, 93.24; specificity, 79.52), respectively (**Table 5** and **Figure 1**).

**Table 5:** Comparison of the biomarkers for predicting the prognosis of COVID-19 patients using ROC analysis.

Variables	AUC	Youden index	95%CI	Cutoff	Sensitivity	Specificity	*P* value
Lymphocytes, ×10^9^/L	0.850	0.598	0.820-0.878	0.78	83.78	75.98	**<0.001**
NLR	0.896	0.682	0.869-0.919	6.28	86.30	81.94	**<0.001**
CRP, mg/L	0.870	0.623	0.840-0.896	42.30	86.30	76.00	**<0.001**
PT,sec	0.858	0.562	0.828-0.885	13.80	85.14	71.08	**<0.001**
D-dimer,μg/L	0.902	0.668	0.875-0.924	0.82	94.59	72.20	**<0.001**
DLR	0.924	0.728	0.900-0.943	1.29	93.24	79.52	**<0.001**

COVID-19, Coronavirus disease 19; ROC,receiver operating characteristics curve; AUC, area under the curve; CI, confidence interval; NLR, neutrophils-to-lymphocytes ratio; CRP, C-reactive proteins; PT, prothrombin time; DLR, D-dimer-to-lymphocytes ratio.

*P* < 0.05 was considered statistically significant. Statistically significant values are showed in bold.

**Figure 2:**
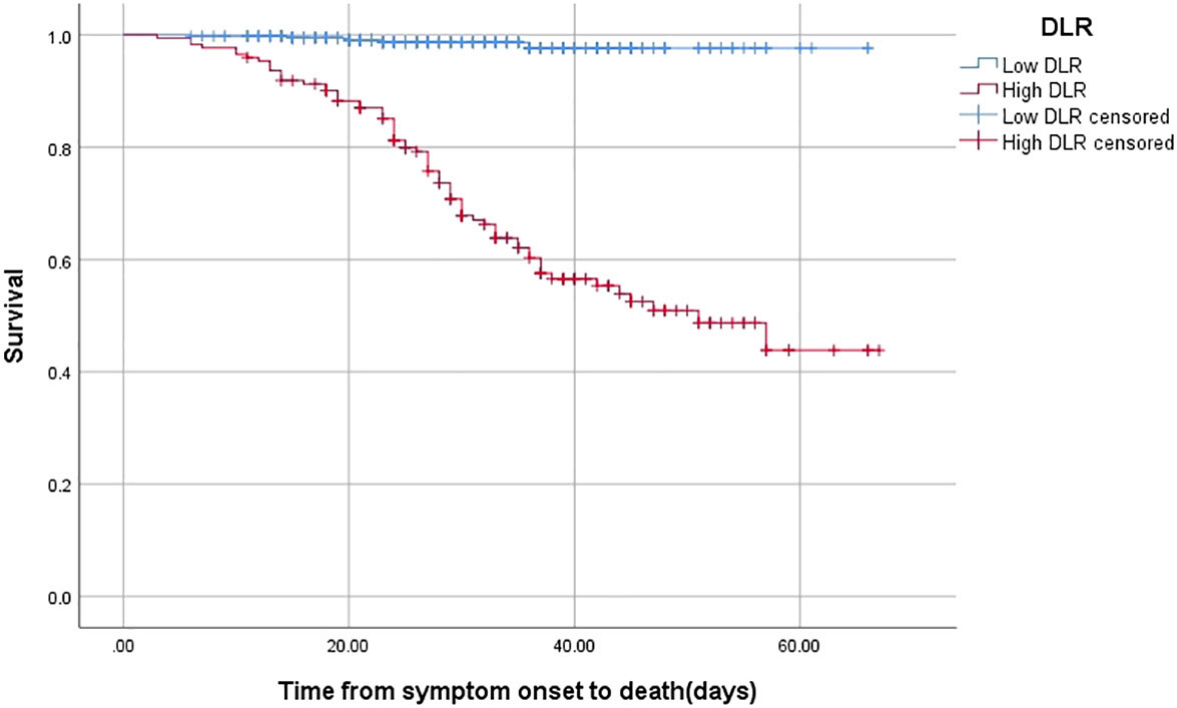
The time-dependent risk of death in COVID-19 patients with low and high DLR using Kaplan-Meier curve. Patients with high DLR showed a worse survival compared with those with low DLR (Log Rank *P <*0.001).

By using the KM curve to investigate the relationship between DLR and survival, patients with high DLR showed a worse survival than those with low DLR (log rank *p* < 0.001) (**Figure 2**).

## Discussion

In the presentinvestigation, we discovered that DLR was a unique risk factor and had the most valuable prediction performance for mortality of in-hospital patients with COVID-19. Additionally, survival rates were worse for individuals with COVID-19 who had higher DLR than for those who had lower DLR.

Recent studies have uncovered that D-dimer was correlated with the severity and prognosis of COVID-19 (Gao et al., 2020; Zhou et al., 2020). Patients with COVID-19 who are hospitalized frequently have coagulopathy. Critically ill COVID-19 patients first exhibit high D-dimer values, which are then followed by an increase in partial thromboplastin times, aberrant prothrombin levels, and lastly low platelet counts (Connors and Levy, 2020). Diffuse alveolar damage, hyaline membrane formation, and pulmonary micro-emboli are highly reported in the autopsy of COVID-19 patients (Fox et al., 2020). In fact, severe acute respiratory syndrome coronavirus 2 (SARS-CoV-2)-induced endothelial dysfunction, platelet activation, excessive inflammation, and stasis can make patients more susceptible to thrombotic arterial and venous occlusions (Piazza et al., 2020). According to Tang et al. (Tang et al., 2020), inflammatory cytokines can disrupt the equilibrium between fibrinolysis and coagulation, and then activate the fibrinolysis system, which raises the level of D-dimer. D-dimer binding to “intracellular” adhesion molecule 1 (ICAM-1), an immunoglobulin-family adhesion molecule that is expressed on the membrane of leukocytes as well as endothelial cells, can increase vascular tone and impair blood circulation (Lominadze et al., 2005).

By increasing blood viscosity and activating signaling pathways dependent on hypoxia-inducible transcription factor, SARS-CoV-2 infection that results in organ damage and hypoxemia may promote the formation of thrombosis (Tang et al., 2020). Furthermore, patients with COVID-19 are more likely to have comorbid conditions, be immobile, and undergo invasive procedures, all of which raise the risk of developing thrombotic events (Zhang et al., 2020). It was reported that the level of D-dimer presented a heightened risk of cardiovascular and peripheral arterial diseases (Soomro et al., 2016). Mohamed et al. (Abu-Farha et al., 2020) genotyped the genes of COVID-19 patients for single nucleotide polymorphisms (SNPs) and found that the top three genetic associations with the elevated level of D-dimer were rs13109457-A, rs12029080-G, and rs6687813-A located in F5, FGG, and FGA, which could aid in identifying patients who are most at risk of thromboembolic complications. It is interesting to note that D-dimer may contribute to the pathophysiology in addition to serving as a biomarker. After administering the purified human fragment D to a rabbit, several physiological events take place. These include hypoxia, complement activation, neutropenia chemotaxis, enhanced pulmonary capillary leakage, platelet aggregation, and prostaglandin synthesis (Manwaring and Curreri, 1981; Manwaring and Curreri, 1982).

In most cases, the immunological response brought on by SARS-CoV-2 infection can be subdued through attracting monocytes and macrophages to the site of infection, releasing cytokines, and initiating adaptive T and B cell immune responses (Tay et al., 2020). However, a defective immune response can occur in specific circumstances, and lymphopenia is a prevalent clinical symptom. Together with profound lung lesions, autopsy results reveal cell degeneration, lymphopenia and necrosis in the spleen, lymphopenia and localized necrosis in the lymph nodes, and decreased myelopoiesis in the bone marrow (Yao et al., 2020).

There are multiple reasons that have been proposed to demonstrate the relationship between lymphopenia and COVID-19. Angiotensin-converting enzyme 2 (ACE2) is the necessary entry receptor for SARS-CoV-2, and the serine protease TMPRSS2 facilitates the interaction of the spike (S) glycoprotein of coronaviruses with receptor molecules (Hoffmann et al., 2020). It has been established that decreased levels of cytotoxic lymphocytes, such as CD8+ T cells and NK cells, had a striking link with elevated levels of ACE2 and TMPRSS2, inferring that patients who are more defenseless against SARS-CoV-2 infection may not have the lung capacity to generate a rapid antiviral cellular immune response (Duijf, 2020).

Immunosenescence is the age-associated functional deterioration of the immune system which includes decreased naïve lymphocytes production, an increase in memory lymphocytes, lower proliferative capacity and impaired function of effector lymphocytes, lymph node fibrosis, and dysregulated cytokine production (Pera et al., 2015). Patients with this condition demonstrate a decreased response to vaccination and are more prone to infection as a result, making the disease more contagious. Likewise, due to underlying comorbidities, aged people with COVID-19 have a higher prevalence of serious diseases and a worse prognosis (Qin et al., 2020). Tumor necrosis factor receptor superfamily member Fas (CD95), which is primarily expressed on effector and memory T cells following interaction with antigen but not on naive T cells, mediates apoptosis *via* death receptor pathway to maintain immunological homeostasis. Bellesi et al. (Bellesi et al., 2020), using flow cytometry, discovered that COVID-19 patients had greater levels of CD95 than controls, and that increased CD95 expression in CD4+ cells was related with decreased CD4+ counts, revealing a further mechanism for lymphopenia by which CD95 triggered apoptosis in COVID-19 patients. Local or systemic cytokine release is typical with COVID-19 and interleukin (IL)-6, IL-1β, and tumor necrosis factor-α (TNF-α) play key roles in the progression and exacerbation pathogenesis of COVID-19. Autophagy, an evolutionarily conserved lysosomal degradation system, has been implicated in regulating maturation and survival of T and B lymphocytes (Vomero et al., 2020). Recently, IL-6, IL-1β, and TNF-α have been described to be involved in autophagy regulation. It has been shown that IL-6 could activate STAT3 through Janus-activated kinases (Jak) (Wang et al., 2013), and rheumatoid arthritis patients treated with anti-Jak inhibitors *in vitro* saw an inhibition of lymphocyte autophagy (Vomero et al., 2020). Tumor necrosis factor-α can induce autophagy, further activate lymphocytes, and attract lymphocytes causing excessive inflammation of typical COVID-19 infection (Vomero et al., 2020). It is widely known that IL-1β has a direct effect on lymphocytes. Inflammasome promotes IL-1β secretion *via* the activation of caspase-1, which medicates autophagy in the regulation of inflammation (Vomero et al., 2020). A novel therapeutic approach for COVID-19 is drugs that target autophagy. Moreover, TNF-α combining with TNFR1 to promote T cell apoptosis is a characteristic of aged T cells (Gupta et al., 2005), IL-2 is an essential cytokine for the proliferation, differentiation, and function of T cells (Ross and Cantrell, 2018), and secretion of cytokines can draw T lymphocytes from blood to the site of infection, which may explain lymphopenia in patients with COVID-19 (Xu et al., 2020). It was observed that after two years of recovery, coronavirus-specific memory T cells could be discovered (Yang et al., 2006).

Hemostasis intricately interacts with the immune system. Neutrophils, platelets, and the coagulation cascade work together to increase the host’s ability to defend itself against pathogenic assaults in immunothrombosis, which is a type of physiological thrombosis (Massberg et al., 2010). The microvasculature is primarily affected by immunothrombotic dysregulation, which can occur in some specific inflammatory situations and upset the natural immunothrombosis equilibrium. Patients with COVID-19 are thought to have this important mechanism (Nicolai et al., 2020). Nicolai et al. (Nicolai et al., 2020) investigated the contribution of immunothrombosis in COVID-19 using immunofluorescence and confocal microscopy and discovered that neutrophils embedded in the fibrin clot in the microthrombi, and numerous intravascular neutrophils were intimately affiliated to platelets and fibrin.

Indeed, circulating platelet-leukocyte complexes can trigger the onset of neutrophil extracellular traps (NETs), which enable the development of immunothrombosis (Clark et al., 2007). Brinkmann et al. (Brinkmann et al., 2004) first described neutrophil cell death in 2004, positing that it could operate as a primitive defense to trap and kill microbial invaders and implement a procoagulant and prothrombotic scaffold (Fuchs et al., 2010). Multiple researchers have revealed that NETs were crucial components of microvascular and macrovascular thrombosis (Blasco et al., 2020) and bronchoalveolar lavage fluid in COVID-19 patients (Ouwendijk et al., 2021). Compared with healthy controls, there was a 50-fold increase in NETs release by neutrophils of COVID-19 patients, and healthy neutrophils might also develop NETs when exposed to plasma from COVID-19 patients (Middleton et al., 2020). In COVID-19, immunothrombotic occlusions with NETs were four times more common than influenza pneumonia (Nicolai et al., 2021). Dexamethasone and colchicine—NETs inhibitors—were recommended to lower COVID-19 patient mortality (Deftereos et al., 2020; Tomazini et al., 2020), and DNase1 could degrade NETs to reduce thrombosis in immunothrombotic models (Jimenez-Alcazar et al., 2017), highlighting that NETs might just be a focus of attention for COVID-19 treatment.

In the study, DLR had a greater AUC than D-dimer or lymphocytes separately for COVID-19 death, which suggested that the combination of D-dimer and lymphocytes might be superior in reflecting the status of COVID-19 patients. We speculated that DLRmight be concerned in the immunothrombosis of COVID-19. To pinpoint the precise mechanism through which DLR contributes to immunothrombosis, more study is necessary.

The study includes several limitations. First, because it was a retrospective study, only the initial level of DLR upon admission was noted and the association between time-dependent changes in DLR and prognostic relevance is yet unknown. Second, the sample size is relatively small, which could have influenced the statistical findings. Third, there was no D-dimer and lymphocyte values prior to COVID-19. For the value of underlying comorbidities with elevated D-dimer levels, this is an important factor to consider. Finally, the study cohort was entirely Chinese, therefore extrapolating the findings to other racial or ethnic groups might be limited.

Taken together, DLR might be quite important in forecasting the outcome of COVID-19 when taken as a whole. To identify viable strategies for treating COVID-19, the levels of DLR should, if necessary, be monitored, and the reason for the change in DLR should be examined.

## Data availability statement

The raw data supporting the conclusions of this article will be made available by the authors, without undue reservation.

## Author contributions

YZ and SW contributed to the conception, study design, implementation, and critical revision. FP contributed to methodology, software, and writing original draft preparation. CL, QY, JD and QZ collected and interpreted the data. MX and CW contributed to the conception and study design. All authors contributed to the article and approved the submitted version.
